# Associations of White Matter Microstructure with Clinical and Demographic Characteristics in Heavy Drinkers

**DOI:** 10.1371/journal.pone.0142042

**Published:** 2015-11-03

**Authors:** Mollie A. Monnig, Ronald A. Yeo, J. Scott Tonigan, Barbara S. McCrady, Robert J. Thoma, Amithrupa Sabbineni, Kent E. Hutchison

**Affiliations:** 1 Center for Alcohol and Addiction Studies, Brown University, Providence, Rhode Island, United States of America; 2 Department of Psychology, University of New Mexico, Albuquerque, New Mexico, United States of America; 3 Center on Alcoholism, Substance Abuse, and Addictions, Albuquerque, New Mexico, United States of America; 4 Department of Psychiatry and Behavioral Sciences, University of New Mexico, Albuquerque, New Mexico, United States of America; 5 Department of Psychology and Neuroscience, University of Colorado at Boulder, Boulder, Colorado, United States of America; UCLA, UNITED STATES

## Abstract

Damage to the brain’s white matter is a signature injury of alcohol use disorders (AUDs), yet understanding of risks associated with clinical and demographic characteristics is incomplete. This study investigated alcohol problem severity, recent drinking behavior, and demographic factors in relation to white matter microstructure in heavy drinkers. Magnetic resonance imaging (MRI) scans, including diffusion tensor imaging (DTI), were collected from 324 participants (mean age = 30.9 ± 9.1 years; 30% female) who reported five or more heavy drinking episodes in the past 30 days. Drinking history and alcohol problem severity were assessed. A common white matter factor was created from fractional anisotropy (FA) values of five white matter tracts: body of corpus callosum, fornix, external capsule, superior longitudinal fasciculus, and cingulate gyrus. Previous research has implicated these tracts in heavy drinking. Structural equation modeling (SEM) analyses tested the hypothesis that, after controlling for duration of alcohol exposure, clinical and behavioral measures of alcohol use severity would be associated with lower white matter factor scores. Potential interactions with smoking status, gender, age, treatment-seeking status, and depression or anxiety symptoms also were tested. Controlling for number of years drinking, greater alcohol problem severity and recent drinking frequency were significantly associated with lower white matter factor scores. The effect of drinking frequency differed significantly for men and women, such that higher drinking frequency was linked to lower white matter factor scores in women but not in men. In conclusion, alcohol problem severity was a significant predictor of lower white matter FA in heavy drinkers, after controlling for duration of alcohol exposure. In addition, more frequent drinking contributed to lower FA in women but not men, suggesting gender-specific vulnerability to alcohol neurotoxicity.

## Introduction

White matter damage is a hallmark injury of alcohol use disorders (AUDs), with substantial atrophy found in postmortem and neuroimaging studies [1, 2]. Animal models of excessive alcohol intake have shown that alcohol neurotoxicity directly damages white matter microstructure [3]. Diffusion tensor imaging (DTI), an application of magnetic resonance imaging (MRI) that measures the mobility of water molecules in tissue, allows for fine-grained, noninvasive examination of white matter microstructure in the human brain [4]. Fractional anisotropy (FA) is a DTI metric that indicates the extent to which diffusion in a voxel is non-random (i.e., anisotropic), and higher FA in white matter generally reflects healthy myelination and organization of fibers. In experimental studies, DTI metrics correspond to axonal integrity and myelination [5, 6].

**Fig 1:**
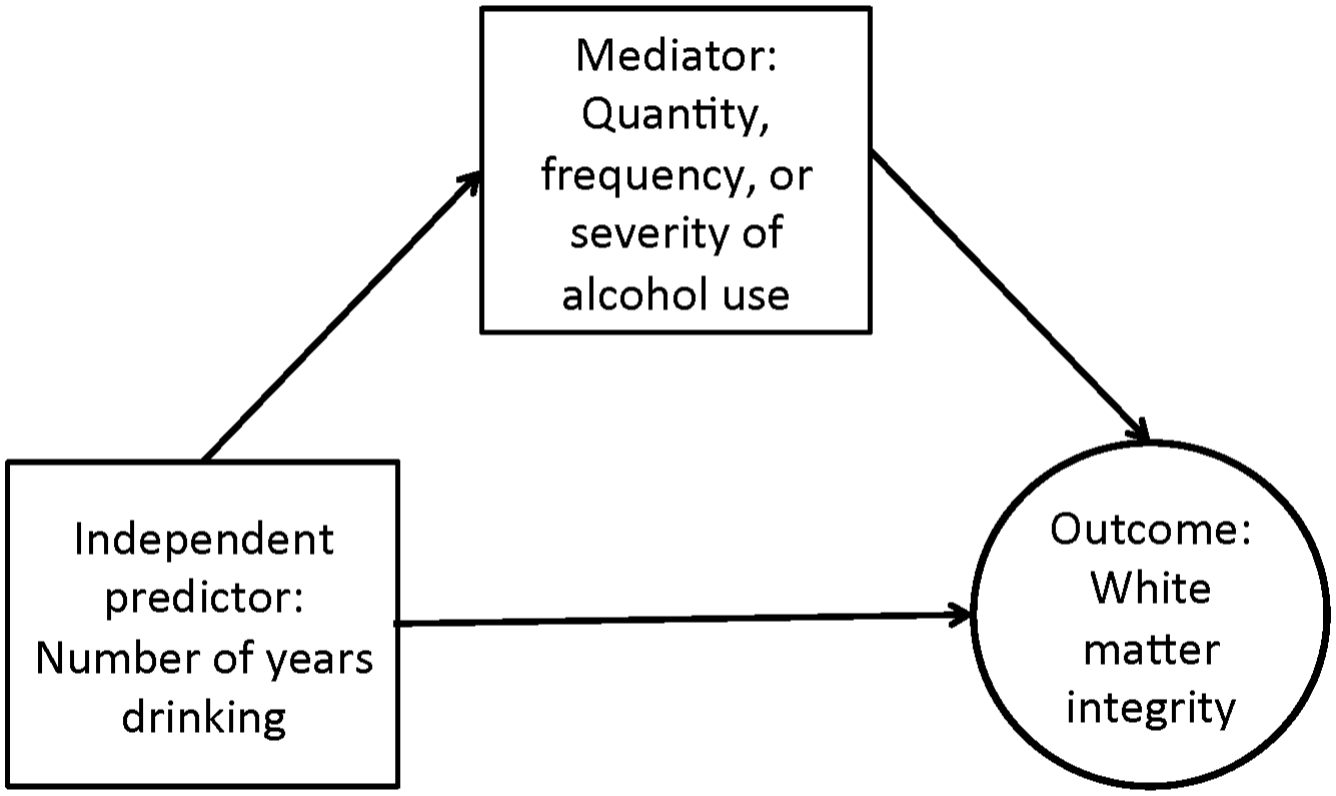
Theoretical model. The model proposes that the direct effect of number of years drinking on white matter integrity is mediated by quantity, frequency, and severity of alcohol use. In model diagrams, squares are observed variables and the circle represents a latent variable.

Numerous DTI studies of individuals with AUDs have found white matter abnormality in networks associated with reward and self-regulation [7–9]. White matter abnormality plays a central role in models of AUDs that propose an imbalance between neural systems of cognitive control and reward-seeking, leading to executive dysfunction, disinhibition, and impaired insight [10]. The aim of the current study was to investigate the impact of chronic alcohol exposure on white matter microstructure as a function of alcohol problem severity, recent drinking behavior, and demographic characteristics. We hypothesized that greater duration of alcohol exposure would predict lower white matter microstructural integrity, and that this association would be mediated by alcohol use quantity, frequency, and severity (Fig 1).

In a recent study, our group found that neural reactivity to an alcohol taste cue in heavy drinkers was associated with FA of several white matter tracts in frontoparietal and corticolimbic networks [11]. Lower white matter FA values in anterior corona radiata, body of corpus callosum, cingulate gyrus, external capsule, fornix, inferior fronto-occipital fasciculus, posterior corona radiata, retrolenticular limb of internal capsule, and superior longitudinal fasciculus were significantly correlated with greater fMRI response to the cue. Comparing those results with previous DTI reports in the literature [7–9, 12], five tracts consistently have been implicated in AUDs: body of corpus callosum, fornix, external capsule, cingulate gyrus, and superior longitudinal fasciculus. These tracts serve numerous, distributed higher-order functions, including visuospatial and language processing (superior longitudinal fasciculus), interhemispheric communication (corpus callosum), limbic processing of emotion and memory (fornix), decision-making and performance monitoring (cingulum), and possibly sensory integration (external capsule) [13–17]. It is possible that lower white matter integrity in these tracts compromises neural networks that regulate response to alcohol, resulting in greater reactivity to alcohol cues.

Although white matter abnormality in AUDs is well established, attempts to link it with specific indices of problem severity, drinking behavior, or demographic characteristics have yielded inconsistent results. Effects of heavy alcohol consumption on white matter may interact with gender, age, treatment-seeking status, smoking status, or comorbid psychiatric disorder. Some studies have found greater alcohol-related white matter damage in women [18], older individuals [12], smokers [19], or treatment-seeking individuals [20]. Other studies have reported null findings with respect to group differences or have found effects in the opposite direction, such as less severe white matter atrophy in women with AUDs [21]. Because the AUD population has elevated rates of depression and anxiety [22], comorbid psychopathology also warrants investigation as a potential moderator.

The present study used structural equation modeling (SEM) to test associations of white matter microstructure with clinical and demographic variables. A latent white matter factor (WMF) was created from FA values of the five tracts of interest. This approach had the benefit of limiting the number of statistical tests by reducing several FA values to a single measure of white matter microstructure. In addition, a latent variable isolates variance common to the observed variables and explicitly adjusts for random error, avoiding the problematic assumption of the absence of measurement error in multiple regression [23, 24]. Mediation analyses are an asset in behavioral research because they allow for testing of explicit hypotheses about how specific variables are related [25].

This study investigated three main research hypotheses. First, we hypothesized that a common factor would account for shared variance in FA values of white matter tracts previously implicated in AUD research (superior longitudinal fasciculus, corpus callosum, cingulate gyrus, fornix, and external capsule), within a heavy-drinking sample. This hypothesis was tested using confirmatory factor analysis (CFA) on the WMF model. Second, we hypothesized that, after controlling for duration of alcohol exposure, clinical and behavioral measures of alcohol problem severity would be associated with lower FA. Number of years drinking was chosen as the independent predictor because our theoretical model (Fig 1) stipulates that duration of alcohol exposure exerts direct neurotoxic effects on white matter microstructure, and these effects are mediated by alcohol use quantity, frequency, and severity. Mediation models asked which clinical features of alcohol use best predicted WMF scores, controlling for number of years drinking. The third hypothesis was that the strength of association between alcohol use variables and white matter integrity would differ as a function of age, gender, treatment-seeking status, smoking status, or comorbid depression/anxiety. Analyses tested whether these group membership variables were significant moderators of mediation effects. Because data were cross-sectional, results of path analyses should be interpreted without inferences about causality.

## Materials and Methods

### Participants

Participants were ages 21–56 years and reported at least 5 heavy drinking episodes (≥ 4 drinks for women, ≥ 5 drinks for men [26]) in the past month. Inclusion and exclusion criteria are described in further detail in our previous study [11]. Briefly, participants were recruited from the community through online, radio, and print advertisements. Both treatment-naïve and treatment-seeking individuals were recruited. Treatment-naïve participants were not seeking treatment at the time of recruitment and had no history of alcohol or drug treatment. Treatment-seeking participants had not yet initiated treatment for alcohol use at the time of the data collection reported here. Current or past AUD diagnosis was not an exclusion criterion for either group. Duration of abstinence was determined via self-report. Participants were required to have a breath alcohol concentration of 0.00 prior to the scan session. Individuals who showed signs of acute withdrawal (CIWA score > 8 [27]) were excluded from participation. All research involving human participants was approved by the Institutional Review Board of the University of New Mexico. Written informed consent for study procedures was obtained from all participants.

Of the 336 participants in Monnig et al. (2013), 12 had outlier FA values > 3 standard deviations (SDs) from the mean and were excluded from the present study. Consequently, 324 participants were included in confirmatory factor analysis (CFA) of the white matter common factor. SEM path analyses were limited to 303 participants who had data for years drinking. Sample characteristics are shown in Table 1. Sample characteristics sorted by moderator variables are shown in S1 Table.

**Table 1:** Participant characteristics.

	*n*	*M*	*SD*
Age	303	30.86	9.08
Sex (% male)	303	70	—
Education (years)	269	14.40	2.51
Years drinking	303	11.70	8.77
ADS score	283	13.11	8.05
AUDIT score	283	18.52	7.65
ICS score	286	44.74	21.00
DPDD	278	7.11	4.29
PDD	278	.59	.25
PHDD	242	.44	.28
Days since last drink	234	2.55	3.07
BDI score	243	11.87	9.42
BAI score	273	9.40	8.36

Abbreviations: ADS = Alcohol Dependence Scale; AUDIT = Alcohol Use Disorder Identification Test; ICS = Impaired Control Scale; DPDD = drinks per drinking day; PDD = proportion of days drinking; PHDD = proportion heavy drinking days; BDI = Beck Depression Inventory; BAI = Beck Anxiety Inventory.

### Measures

Participants completed the Alcohol Use Disorders Identification Test (AUDIT [28]), the Alcohol Dependence Scale (ADS [29]) and the Impaired Control Scale (ICS [30]), as well as demographic and drinking history questionnaires. Total scores from the ADS, AUDIT, and ICS were used in analyses. Number of years drinking was calculated by subtracting the age at which the individual began drinking regularly, defined as “at least once a month for six months or more,” from current age. Recent alcohol use and smoking were assessed using the Timeline Followback [31]. Drinks per drinking day (DPDD), proportion of days drinking (PDD), proportion heavy drinking days (PHDD), and number of days since last drink were chosen as measures of quantity, frequency, and recency. PDD and PHDD were calculated over the past 60 or 90 days, using the definition of heavy drinking above. Clinical symptoms of depression and anxiety were assessed with the Beck Depression Inventory-II (BDI [32]) and Beck Anxiety Inventory (BAI [33]), respectively.

Age groups were formed using a median split (median age = 27.5 years). Participants were coded as smokers if they reported any smoking on the Timeline Followback. Smokers reported an average of 8.5 ± 7.5 cigarettes per smoking day. A dichotomous variable (0 = low, 1 = high) was created to indicate the presence of significant comorbid anxiety or depression according to published clinical cutoffs on the BAI and BDI. Participants with BAI score > 15 or BDI score > 13 were coded as high comorbidity. Interest in comorbidity pertained to the presence of common symptomatology rather than either specific diagnosis. For 202 participants with scores for both assessments, the BDI and BAI were moderately correlated (*r* = 0.470, *p* < 0.001).

Diagnosis of AUD according to the Diagnostic and Statistical Manual of Mental Disorders (DSM) or other formal system was not available for most participants. To facilitate comparison with previous studies of individuals with confirmed AUDs, we report percentages of the sample endorsing high levels of alcohol problems on widely used screening instruments, the ADS and AUDIT. The percentage of the sample meeting the threshold for high probability of an AUD, with a score of 9 or higher, was 64% on the ADS [34]. The percentage meeting criteria for high probability of an AUD, with a score of 16 or higher, was 58% on the AUDIT [28].

### DTI variables

**Fig 2:**
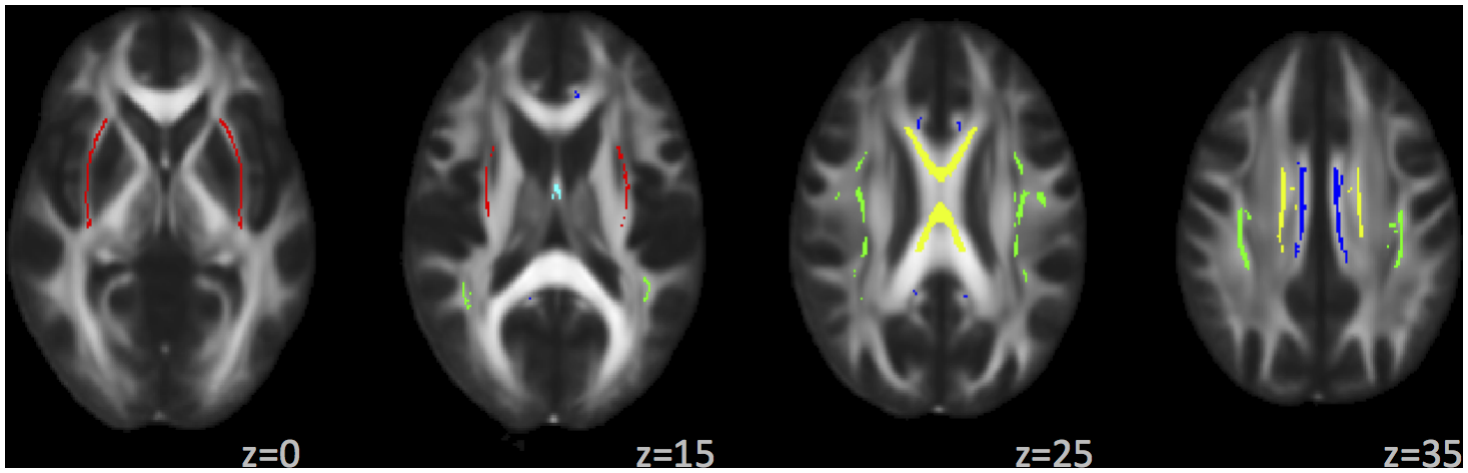
Tracts included in the White Matter Factor (WMF), shown on FSL’s FMRIB58 FA image. Color legend: external capsule, red; fornix, light blue; body of corpus callosum, yellow; cingulate gyrus, dark blue; superior longitudinal fasciculus, green.

MRI scans were obtained on 3T Siemens Trio scanner. Localizer scans were acquired with an echo-planar, gradient-echo, pulse sequence (TR = 2000 ms, TE = 29 ms, flip angle = 75°) with a 12-channel head coil, parallel to the ventral surface of the participant’s orbitofrontal cortex. Each volume consisted of 33 axial slices (64x64 matrix, 3.75x3.75 mm^2^, 3.5 mm thickness, 1 mm gap). High-resolution T1-weighted MP-RAGE anatomical scans were acquired with TR = 2530 ms, TE = 1.64 ms, flip angle = 7°, 192 sagittal slices, 256x256 matrix, slice thickness = 1 mm). DTI scans were acquired with single-shot, spin-echo, echo-planar imaging along the AC/PC line with FOV = 256x256 mm, 128x128 matrix, slice thickness = 2mm (isotropic 2 mm resolution), NEX = 1, TE = 84 ms, and TR = 9000 ms. A multiple-channel radiofrequency channel coil was used with GRAPPA(X2), 30 gradient directions, *b* = 800 s/mm^2^, and *b* = 0 repeated 5 times. DTI preprocessing steps included quality check to exclude volumes with scanner noise, signal dropout, or excessive motion; motion eddy current correction; and adjustment of diffusion gradient directions. A DTI volume was excluded if the motion was more than 4 mm of root mean square displacement, and a subject’s dataset was excluded if more than 10% of gradient directions were dropped for any reason. All images were registered to a *b* = 0 s/mm^2^ image using 12 degrees of freedom, affine transformation with mutual information cost function. Image registration and transformation steps were performed with the FMRIB Software Library (FSL) Linear Image Registration Tool. Outlier detection and data pruning were done with a custom program written in IDL (www.ittvis.com). Dtifit was used to calculate diffusion tensor and FA maps. FA values were obtained using FMRIB’s tract-based spatial statistics [35]. FA images were aligned to the standard-space FMRIB58 FA image (1x1x1 mm) using FMRIB’s Nonlinear Image Registration Tool. After transformation to the target and affine transformation to MNI152 space, all FA images were merged into a single 4D image file from which the FA skeleton was calculated using a threshold value of 0.2. White matter tracts were defined using the Johns Hopkins University International Consortium for Brain Mapping (JHU-ICBM) DTI-81 atlas with highest probability thresholding at 25%. An FA value for each tract was obtained by averaging over voxels of the white matter skeleton located within that tract. FA values for the entire sample are shown in Table 2 and according to subgroup in S1 Table. Fig 2 shows the skeletonized white matter tracts included in WMF.

**Table 2:** FA values for tracts included in the white matter factor (WMF).

	*M*	*SD*
Body of corpus callosum	0.72	0.03
Fornix	0.49	0.07
External capsule, right	0.47	0.02
External capsule, left	0.51	0.03
Cingulate gyrus, right	0.60	0.04
Cingulate gyrus, left	0.66	0.04
Superior longitudinal fasciculus, right	0.51	0.03
Superior longitudinal fasciculus, left	0.55	0.03

### Statistical analysis

#### Data transformations

Square-root transformation was applied to DPDD to reduce skewness. Because transformation did not normalize the distribution for days since last drink, ten outliers of > three SDs were excluded from those analyses. Following Mplus recommendations [36], FA values were rescaled by multiplying by 100, so that variables in the model were on similar scales and FA variances ranged from 1–10.

#### Confirmatory factor analysis (CFA)

Analyses were conducted on the variance-covariance matrix (S2 Table) in Mplus version 7 [36] using maximum likelihood estimation (ML). The latent variable was scaled by setting its variance to 1.0. The model allowed correlated errors between right- and left-hemisphere counterparts of the bilateral tracts and between the two midline tracts (body of corpus callosum and fornix). Correlated errors are appropriate when part of the covariation between two indicators is attributable to sources other than the common factor, such as source effects or method effects [24]. Tract-based spatial statistics procedures are reliable yet can produce systematic errors in steps of image registration and/or skeleton projection [37], which would be expected to correlate for left/right pairs and midline structures. Other measurement error was presumed to be uncorrelated. Bivariate Pearson correlations for FA values (excluding left and right pairs) were moderate (average *r* = .41; range = .12-.56), showing that values were not so highly correlated as to be redundant.

The significance value for factor loadings was set to *p* < 0.05. For evaluation of model fit, standardized root mean square residual (SRMR), root-mean-square error of approximation (RMSEA), and comparative fit index (CFI) are reported as absolute, parsimony-corrected, and comparative goodness-of-fit indices, respectively [38]. Following Hu and Bentler (1999), SRMR < 0.08, RMSEA < 0.06, and CFI > 0.95 were interpreted as indicating good model fit. RMSEA < 0.10 and CFI > 0.90 were interpreted as marginally acceptable model fit. Steps taken to minimize Type I error included theory-driven analysis, data reduction to limit number of tests, avoidance of iterative model respecification, and use of multiple fit indices [24, 39].

#### Structural equation models (SEM)

Analyses were conducted on the variance-covariance matrix using ML estimation in Mplus version 7. Standard errors of model parameter estimates, including indirect effects, and their confidence intervals (CIs) were estimated using the bootstrap option with 10,000 iterations. Bootstrapped CIs are a more accurate test of significance for the indirect effect than the traditional *p*-value [40]. Both *p*-values and CIs are reported where relevant.

SEM analyses tested whether alcohol problem severity or recent drinking behavior mediated the path from number of years drinking to WMF. ADS, AUDIT, ICS, PDD, DPDD, PHDD, and days since last drink were tested as mediators. The objective was to identify which variables best predicted WMF, controlling for years drinking. Mediation analyses can shed light on how variables are related by separately quantifying several effects: the independent variable on the mediator (*a* path); the mediator on the dependent variable, controlling for the independent variable (*b* path); the total indirect effect (product of *a* and *b* paths); and the independent variable on the dependent variable, after controlling for the mediator (direct effect).

Number of years drinking was highly correlated with age (*r* = .877, *p* < .001). Age is known to strongly predict change in white matter microstructure in healthy individuals [41, 42]. Age was not included as an independent predictor because its high degree of shared variance with years drinking led to problems of multicollinearity when including both predictors. However, age was tested as a moderator (see below).

Moderated mediation models [43] assessed whether mediation effects differed depending on gender, treatment-seeking status, smoking status, age, or comorbidity. In other words, these models tested whether between-group characteristics showed interactions with effects found in the simple mediation models. As a preliminary step, main effects of group status were tested in repeated measures analysis of variance (ANOVA), with group entered as a between-subjects factor and FA values entered as within-subjects measures.

To limit the number of tests, moderated mediation models used only those mediators that performed well in whole-group analyses. Moderation of the *a* path and *b* path was tested by freeing these parameters one at a time while constraining all other paths in the model to be equal across groups. Factor loadings and intercepts were constrained to be equal for each group, and residual variances were allowed to vary. The *χ* ^2^ difference test evaluated difference in fit between the parent model, in which all paths were constrained to equality, with the nested model with the freed parameter (i.e., the *a* path or *b* path). A significant *χ* ^2^ value is evidence of a significant moderation effect [44].

## Results

### CFA

**Fig 3:**
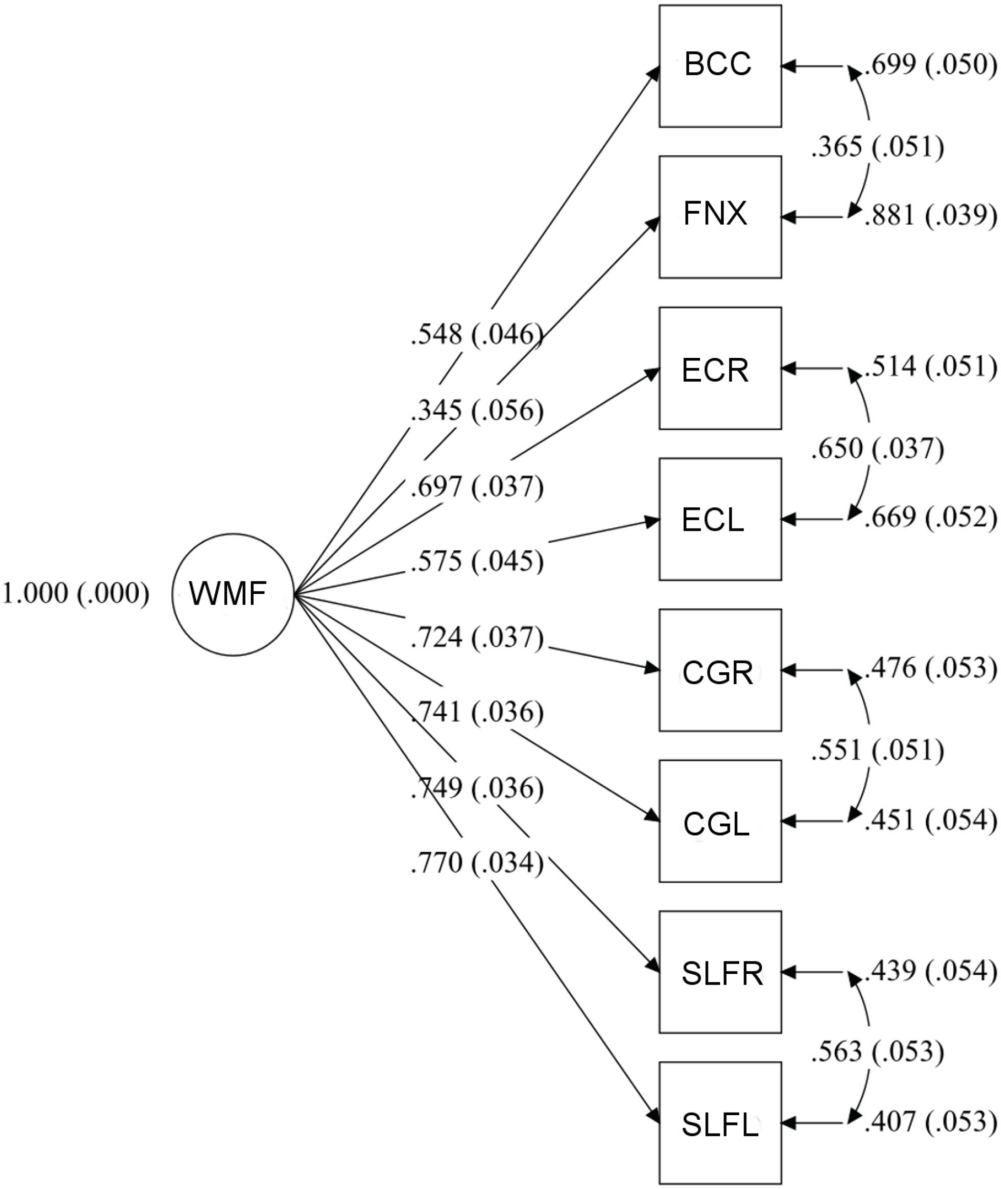
Measurement model of the White Matter Factor (WMF). In all diagrams, the circle signifies the latent variable and squares represent observed variables. Arrows from the latent factor to indicators are factor loadings. In this model, standardized factor loadings can be interpreted as the correlation between the indicator and the latent factor [24]. Arrows drawn to the right of indicators represent error variance, i.e., variance that is unique to the indicator and is not accounted for by the latent variable. Arrows drawn between error variances are correlated errors of measurement, which can arise from source or method effects. Numbers in parentheses are standardized errors. Abbreviations: BCC = body of corpus callosum; CGL = cingulate gyrus, left; CGR = cingulate gyrus, right; ECL = external capsule, left; ECR = external capsule, right; FNX = fornix; SLFL = superior longitudinal fasciculus, left; SLFR = superior longitudinal fasciculus, right.

The WMF model (Fig 3) provided a very good fit to the data, RMSEA = 0.045 (90% CI = 0.004–0.074), CFI = 0.993, SRMR = 0.024. All factor loadings were significant, and the WMF accounted for variance ranging from R^2^ = 0.119 for the fornix to R^2^ = 0.593 for the left superior longitudinal fasciculus.

### Simple mediation

The effect of years drinking on WMF prior to adding mediators was significant (*b* = -0.266, *p* < 0.001). Thus, longer duration of alcohol use was significantly associated with lower WMF scores. When adding mediators, some commonalities were observed. First, the direct effect from years drinking to WMF remained significant after addition of the mediator in all models. Second, the path from years drinking to the mediator (i.e., *a* path) was significant for all variables except last drink. Third, overall model fit was acceptable for each model according to CFI and SRMR and was marginal according to RMSEA. Results are shown in Table 3.

**Table 3:** Results of simple mediation models.

Mediator	*n*	RMSEA	CFI	SRMR	*a* path *p*-value	*b* path *p*-value	Indirect effect *p*-value	Direct effect *p*-value	*β*	*R* ^2^
ADS*	303	0.102	0.939	0.065	< .001	0.008	0.033	0.004	-.176	.100
AUDIT	303	0.105	0.935	0.070	< .001	0.225	0.242	0.003		
ICS	303	0.100	0.942	0.071	< .001	0.111	0.122	0.004		
DPDD	303	0.097	0.944	0.062	< .001	0.178	0.214	0.003		
PHDD	303	0.096	0.946	0.068	< .001	0.590	0.596	0.002		
PDD*	303	0.097	0.944	0.066	< .001	0.034	0.051	0.005	-.145	.090
Last drink	293	0.098	0.942	0.062	0.650	0.315	0.762	0.001		

* indicates significant mediator. For significant mediators, *β* is the standardized coefficient for the regression of WMF on the mediator. The standardized coefficient *β* is the change in the outcome in SD units for one SD change in the predictor. *R* ^2^ is the variance accounted for in WMF by the model.

Abbreviations: ADS = Alcohol Dependence Scale; AUDIT = Alcohol Use Disorder Identification Test; CFI = comparative fit index; DPDD = drinks per drinking day; ICS = Impaired Control Scale; Last drink = days since last drink; PDD = proportion of days drinking; PHDD = proportion heavy drinking days; RMSEA = root-mean-square error of approximation; SRMR = standardized root mean square residual.

ADS and PDD significantly mediated the path from number of years drinking to WMF (i.e., *b* path). The test of the total indirect effect was significant for ADS, and the CI did not include 0 (unstandardized estimate = -0.006; 95% CI = -0.012–-0.002). Although the *p*-value for the indirect effect of PDD was at trend level (*p* = .051), the CI did not include zero (unstandardized estimate = -0.006; 95% CI = -0.012–-0.001), indicating that PDD also was a significant mediator. A multiple mediation model to test mediation by ADS and PDD simultaneously is reported in S1 File.

### Between-group effects and moderated mediation

Preliminary ANOVAs to test effects of group status on observed FA values were significant for treatment-seeking status [*F*(1,322) = 26.715, *p* < .001], gender [*F*(1,322) = 6.528, *p* = .011], age group [*F*(1,322) = 25.200, *p* < .001], and comorbidity [*F*(1,322) = 5.229, *p* = .023], but not for smoking status. FA was significantly lower in treatment-seeking individuals, women, the older age group, and individuals with comorbid anxiety or depression.

**Fig 4:**
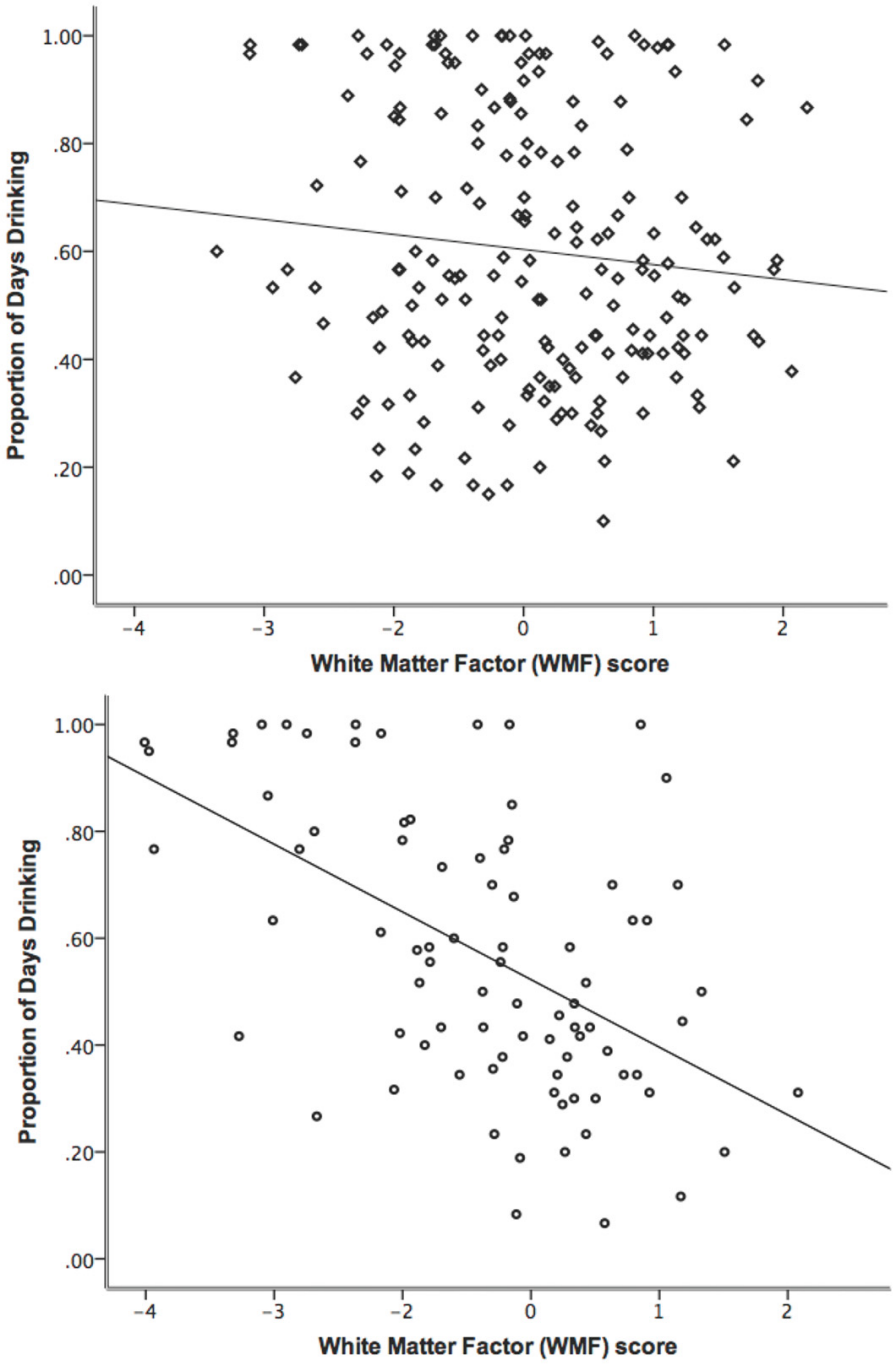
Scatterplots of PDD and WMF score by gender. Data are shown in upper panel for males and in lower panel for females. Gender was a significant moderator of the indirect effect of years drinking to WMF through PDD. *R* ^2^ = .011 for men; *R* ^2^ = .280 for women.

Next, moderation mediation models tested whether mediation effects for ADS or PDD differed by treatment-seeking status, gender, age, smoking status, or comorbidity. Gender significantly moderated the path from PDD to WMF (i.e., *b* path; *χ* ^2^ difference test *p*-value = .002). The path from PDD to WMF was significant for women (standardized *β* = -.417, *p* < .001) but not for men (*β* = -.019, *p* = .811), meaning that frequency of drinking had a negative effect on WMF scores only in women (Fig 4). Because some tracts normally exhibit sexual dimorphism, a post hoc analysis compared the magnitude of the independent effects of gender and PDD on WMF. In a multiple regression analysis with gender (coded male = 1, female = 2) and PDD as independent predictors, no mediators, and WMF as the outcome, both predictors were significant (gender: *β* = -.209, *p* = .001; PDD: *β* = -.203, *p* = .001). Tests of other between-group factors as moderators did not find evidence that mediation effects differed by group.

## Discussion

This study had three main findings related to white matter microstructure in heavy drinkers. First, we hypothesized that a single factor would account for covariance in FA values of the corpus callosum, external capsule, cingulate gyrus, superior longitudinal fasciculus, and fornix. The finding that a common factor (WMF) provided a very good fit to DTI data from these tracts supported this hypothesis. Second, as predicted, individuals with greater alcohol problem severity or drinking frequency had lower white matter FA, after controlling for duration of alcohol exposure. Specifically, problem severity (ADS score) and drinking frequency (recent PDD) significantly mediated the path from number of years drinking to WMF. Third, the effect of PDD on white matter FA differed by gender, such that more frequent drinking was associated with lower FA in women but not in men. Our previous study on this sample of heavy drinkers found that lower FA in these white matter tracts correlated with greater neural reactivity to an alcohol cue. A contribution of the current study is to link drinking behavior, clinical characteristics, and demographic factors to white matter microstructure of these tracts.

The tracts included in the WMF were selected using a combination of *a priori* and data-driven methods. The common factor reflected shared variance in FA values of the tracts used as its indicators and therefore was a specific, rather than global, representation of white matter microstructure. These tracts have been implicated in the pathophysiology of AUDs, and our analysis was a novel approach to investigate associations between white matter microstructure and clinical characteristics in heavy drinkers. Previous neuroimaging studies of AUDs have demonstrated the ability of heavy drinking to damage brain tissue, yet specific markers of risk have yet to be identified [45]. A clinically relevant finding of this study is to suggest potential behavioral markers of risk for white matter abnormality in heavy drinkers. Compared to other drinkers with a similar duration of regular drinking, an individual with a higher ADS score may be at greater risk for white matter damage.

Higher PDD also was linked with lower white matter FA, and this effect was significantly moderated by gender. Women, but not men, showed a significant relationship between greater drinking frequency and lower white matter factor scores (Fig 4). The moderating effect of gender is compelling in light of the fact that male and female groups were highly similar in demographic characteristics, drinking history, and self-reported problem severity. Only DPDD differed between men and women, with men consuming approximately two more DPDD than women. This difference is on par with typical gender differences in alcohol metabolism and blood alcohol levels [46]. Thus, the moderating effect of gender was not confounded by differences in age, drinking behavior, or problem severity. This finding contributes to the ongoing debate as to whether women and men suffer comparable alcohol-related brain damage, given similar duration and quantity of drinking. It is consistent with a previous study that identified lower FA values in several tracts in females with AUDs compared to males with AUDs after matching groups on length of abstinence and lifetime alcohol consumption [7].

Gender also exerted a main effect, with women having lower FA overall. Many DTI studies have reported sexual dimorphism in healthy individuals. Previously, men have shown higher FA in the bilateral tracts included here, whereas women have less consistently shown higher FA in corpus callosum and fornix [47–49]. In a post hoc multiple regression analysis, both gender and PDD had significant effects on white matter when controlling for the other, suggesting that gender has a direct influence that is independent of alcohol use. This finding warrants further investigation, as study design was not able to conclusively differentiate normal gender differences from alcohol-related neurotoxicity.

The main effect of treatment-seeking status on FA values, wherein treatment-seeking individuals had lower FA compared to treatment-naïve individuals, is consistent with previous reports of greater white matter abnormality in treatment seekers [20]. The absence of interaction effects for treatment-seeking status does not contradict previous findings. The current results extend previous research by showing that associations of ADS or PDD with white matter microstructure did not operate differentially depending on treatment-seeking status. Results suggest that differences are a function of problem severity. Surprisingly, given previous research suggesting that heavy drinking and smoking interact to heighten brain structural or metabolic abnormality [19], smoking status was not a significant moderator. A caveat is that the coarse criterion of any smoking in the past 2–3 months may have failed to capture effects of chronic smoking on white matter microstructure. Moreover, smoking has been linked with both increases and decreases in FA in non-AUD samples, potentially obscuring an overall effect [50–53].

Several limitations should be considered when interpreting study findings. One reason for using number of years drinking as the independent predictor was to relate historical data to current white matter microstructure. Nevertheless, the cross-sectional study design does not account for the likelihood of premorbid differences or allow for causal inferences. Because number of years drinking was highly correlated with age, we were unable to differentiate the effects of age and chronic alcohol exposure on white matter microstructure. We observed a main effect of age on FA in which the older group (ages 27.5–56) had lower FA values overall. Two large-scale studies of healthy white matter development have placed peak FA at approximately age 30 for most tracts [41, 42]. The age range of this sample was limited (21–56 years), and 62% of participants were age 30 or under, making it seem unlikely that age-related FA decline drove results. Age was not a significant moderator, meaning that associations of white matter microstructure with predictors of interest were comparable in older and younger groups. Although the purpose of the study was to evaluate predictors of white matter FA within heavy drinkers rather than to identify differences from non-heavy drinkers, comparison with a control group would be helpful for contextualizing the functional significance of white matter microstructural variation. The statistical approach also carried certain limitations. Use of a common factor precluded testing of tract-specific associations with alcohol use or testing of hemispheric laterality effects. For mediation models, overall model fit was more successful according to the absolute and comparative fit indices than the parsimony-corrected fit index. Given acceptable absolute and comparative fit, we retained the original, theoretically-based models rather than increase the risk of Type I error through post hoc model modification. According to power guidelines [54], the sample size of 303 for path analyses was adequate to detect a medium-sized effect, and so smaller effects may have gone undetected. Results should be considered preliminary pending replication and expansion in an independent sample.

In summary, this study linked alcohol problem severity to white matter abnormality and provided initial evidence of female-specific vulnerability to frequency of heavy drinking. It utilized advanced statistical modeling to address pressing clinical research questions about alcohol use and white matter microstructure. Future studies might benefit from integrating additional DTI metrics and relating white matter microstructure to gray matter structure and function.

## Supporting Information

S1 File Multiple mediation model with ADS and PDD. (PDF)

S1 Table Participant characteristics and FA values by moderator. * indicates a significant difference (*p* < .05) between groups in a moderator category. Pairwise group differences in FA were not the focus of the current study and therefore were not tested. FA values by moderator group are shown only for descriptive purposes. Measure abbreviations: ADS = Alcohol Dependence Scale; AUDIT = Alcohol Use Disorder Identification Test; ICS = Impaired Control Scale; DPDD = drinks per drinking day; NYD = number of years drinking; PDD = proportion of days drinking; PHDD = proportion heavy drinking days. Tract abbreviations: BCC = body of corpus callosum; CGL = cingulate gyrus, left; CGR = cingulate gyrus, right; ECL = external capsule, left; ECR = external capsule, right; FNX = fornix; SLFL = superior longitudinal fasciculus, left; SLFR = superior longitudinal fasciculus, right.
(PDF)

S2 Table Variance-covariance matrix for confirmatory factor analysis of the white matter factor (WMF). (PDF)
