# Supplementary material for: Associations of White Matter Microstructure with Clinical and Demographic Characteristics in Heavy Drinkers
Source: PLoS One. 2015 Nov 3;10(11):e0142042. doi: 10.1371/journal.pone.0142042 (PMC4631485; doi:10.1371/journal.pone.0142042)
Supplement: S1 File — (PDF) [file pone.0142042.s001.pdf]

Multiple mediation model for ADS and PDD

The two significant mediators in simple mediation analyses (ADS and PDD) were tested in a multiple mediator analysis. This analysis tested the association of ADS and PDD with WMF while controlling for the other. It included a contrast to test whether indirect effects for mediators differed from each other [1]. The bivariate correlation between ADS and PDD was significant but small ($r = .211$, $p < .001$). The model (S1 Fig., below) had marginal to acceptable values for model fit, RMSEA = .093, CFI = .937, SRMR = .068. The total indirect effect was significant, $p = 0.005$. The CI around the indirect effect for ADS did not include 0 (unstandardized indirect effect = -0.005; 95% CI = -0.012 – -0.001), indicating significant mediation. The CI for the indirect effect of PDD did include zero, showing that it was not a significant mediator (unstandardized indirect effect = -0.005; 95% CI = -0.012 – 0.000). A *z*-test contrast of whether the indirect effects for ADS and PDD differed in magnitude [40] was non-significant ($p = 0.974$). The variance accounted for by this model, at $R^2 = .110$, was comparable to either simple mediator model.

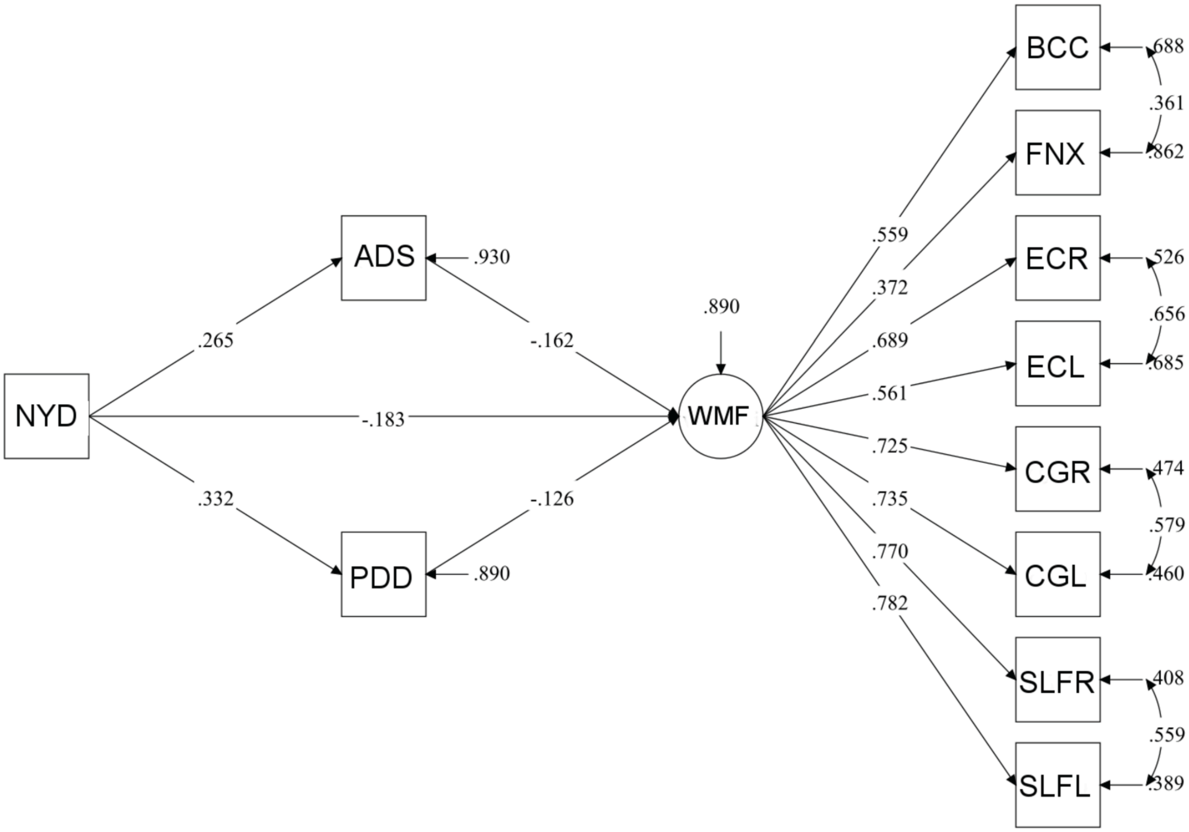

S1 Fig. Partial multiple mediation of the path from years drinking to WMF by ADS and PDD. Abbreviations: ADS = Alcohol Dependence Scale; BCC = body of corpus callosum; CGL = cingulate gyrus, left; CGR = cingulate gyrus, right; ECL = external capsule, left; ECR = external capsule, right; FNX = fornix; PDD = proportion of days drinking; NYD = number of years drinking; SLFL = superior longitudinal fasciculus, left; SLFR = superior longitudinal fasciculus, right.

References

1. Preacher KJ, Hayes AF. Asymptotic and resampling strategies for assessing and comparing indirect effects in multiple mediator models. Behav Res Methods. 2008;40:879-91.
