# Supplementary material for: Associations of White Matter Microstructure with Clinical and Demographic Characteristics in Heavy Drinkers
Source: PLoS One. 2015 Nov 3;10(11):e0142042. doi: 10.1371/journal.pone.0142042 (PMC4631485; doi:10.1371/journal.pone.0142042)
Supplement: S1 Table — * indicates a significant difference (p < .05) between groups in a moderator category. Pairwise group differences in FA were not the focus of the current study and therefore were not tested. FA values by moderator group are shown only for descriptive purposes. Measure abbreviations: ADS = Alcohol Dependence Scale; AUDIT = Alcohol Use Disorder Identification Test; ICS = Impaired Control Scale; DPDD = drinks per drinking day; NYD = number of years drinking; PDD = proportion of days drinking; PHDD = proportion heavy drinking days. Tract abbreviations: BCC = body of corpus callosum; CGL = cingulate gyrus, left; CGR = cingulate gyrus, right; ECL = external capsule, left; ECR = external capsule, right; FNX = fornix; SLFL = superior longitudinal fasciculus, left; SLFR = superior longitudinal fasciculus, right. (PDF) [file pone.0142042.s002.pdf]

**S1 Table. Participant characteristics and FA values by moderator.**

| | Treatment-seeking status | | | | Gender | | | | Smoking status | | | | Age group | | | | Comorbidity | | | |
|---|---|---|---|---|---|---|---|---|---|---|---|---|---|---|---|---|---|---|---|---|
| | Treatment-naïve | | Treatment-seeking | | Male | | Female | | Non-smoker | | Smoker | | Younger | | Older | | Low | | High | |
| | (*n* = 189) | | (*n* = 114) | | (*n* = 211) | | (*n* = 92) | | (*n* = 141) | | (*n* = 161) | | (*n* = 149) | | (*n* = 154) | | (*n* = 185) | | (*n* = 112) | |
| | *M* | *SD* | *M* | *SD* | *M* | *SD* | *M* | *SD* | *M* | *SD* | *M* | *SD* | *M* | *SD* | *M* | *SD* | *M* | *SD* | *M* | *SD* |
| Age | 26.4* | 5.6 | 38.3* | 8.9 | 31.0 | 9.1 | 30.8 | 9.0 | 31.0 | 9.6 | 30.7 | 8.6 | 23.6* | 1.9 | 37.9* | 7.7 | 30.6 | 9.4 | 31.8 | 8.6 |
| % male | 71 | — | 67 | — | 100 | — | 0 | — | 67 | — | 72 | — | 69 | — | 71 | — | 72 | — | 66 | — |
| Education | 15.0* | 2.6 | 13.5* | 2.0 | 14.3 | 2.5 | 14.7 | 2.4 | 14.8* | 2.5 | 14.1* | 2.5 | 14.9* | 2.7 | 13.9* | 2.2 | 14.8* | 2.2 | 13.8* | 2.8 |
| NYD | 8.1* | 5.8 | 17.7* | 9.6 | 12.1 | 9.1 | 10.8 | 8.1 | 11.2 | 8.9 | 12.0 | 8.6 | 5.5* | 2.8 | 17.7* | 8.4 | 11.2 | 8.9 | 12.8 | 8.5 |
| ADS | 10.0* | 5.6 | 17.7* | 8.9 | 12.7 | 7.8 | 14.1 | 8.7 | 11.8* | 7.8 | 14.3* | 8.2 | 10.6* | 6.1 | 15.4* | 8.9 | 10.3* | 6.4 | 17.5* | 8.5 |
| AUDIT | 15.1* | 6.1 | 23.7* | 6.8 | 18.8 | 7.4 | 17.9 | 8.1 | 17.0* | 7.7 | 19.8* | 7.4 | 15.6* | 6.4 | 21.3* | 7.7 | 16.0* | 6.8 | 22.7* | 7.0 |
| ICS | 33.6* | 17.3 | 61.6* | 13.5 | 44.3 | 20.7 | 45.8 | 21.8 | 41.9* | 21.2 | 47.1* | 20.5 | 35.0* | 19.0 | 53.7* | 18.6 | 37.9* | 19.5 | 55.8* | 18.6 |
| DPDD | 5.9* | 2.5 | 8.9* | 5.5 | 7.7* | 4.6 | 5.8* | 3.3 | 6.8 | 4.9 | 7.4 | 3.7 | 5.7* | 2.3 | 8.4* | 5.2 | 6.3* | 3.1 | 8.4* | 5.4 |
| PDD | 0.53* | 0.22 | 0.69* | 0.27 | 0.60 | 0.25 | 0.57 | 0.26 | 0.59 | 0.26 | 0.59 | 0.25 | 0.51* | 0.21 | 0.67* | 0.26 | 0.57 | 0.26 | 0.63 | 0.25 |
| PHDD | 0.34* | 0.20 | 0.58* | 0.32 | 0.45 | 0.29 | 0.41 | 0.28 | 0.41 | 0.29 | 0.45 | 0.27 | 0.32* | 0.20 | 0.56* | 0.31 | 0.40* | 0.28 | 0.49* | 0.28 |
| Last drink | 2.2* | 1.4 | 3.1* | 4.5 | 2.6 | 3.2 | 2.4 | 2.9 | 2.7 | 2.8 | 2.4 | 3.3 | 2.4 | 2.8 | 2.7 | 3.4 | 2.5 | 3.2 | 2.6 | 3.0 |
| BCC | 0.72 | 0.03 | 0.71 | 0.03 | 0.72 | 0.03 | 0.72 | 0.03 | 0.72 | 0.03 | 0.72 | 0.03 | 0.73 | 0.02 | 0.71 | 0.04 | 0.72 | 0.03 | 0.72 | 0.03 |
| Fornix | 0.50 | 0.06 | 0.45 | 0.07 | 0.48 | 0.07 | 0.49 | 0.07 | 0.48 | 0.07 | 0.49 | 0.07 | 0.51 | 0.06 | 0.46 | 0.07 | 0.49 | 0.07 | 0.49 | 0.07 |
| ECR | 0.47 | 0.02 | 0.47 | 0.03 | 0.47 | 0.02 | 0.47 | 0.02 | 0.47 | 0.02 | 0.47 | 0.02 | 0.47 | 0.02 | 0.47 | 0.03 | 0.47 | 0.02 | 0.47 | 0.03 |
| ECL | 0.51 | 0.02 | 0.51 | 0.03 | 0.51 | 0.03 | 0.51 | 0.03 | 0.51 | 0.03 | 0.51 | 0.03 | 0.51 | 0.02 | 0.51 | 0.03 | 0.51 | 0.03 | 0.51 | 0.03 |
| CGR | 0.60 | 0.04 | 0.59 | 0.03 | 0.60 | 0.04 | 0.58 | 0.03 | 0.60 | 0.03 | 0.60 | 0.04 | 0.60 | 0.04 | 0.59 | 0.04 | 0.60 | 0.04 | 0.60 | 0.04 |
| CGL | 0.66 | 0.03 | 0.65 | 0.04 | 0.66 | 0.03 | 0.64 | 0.04 | 0.66 | 0.03 | 0.66 | 0.04 | 0.66 | 0.03 | 0.65 | 0.04 | 0.66 | 0.04 | 0.66 | 0.04 |
| SLFR | 0.52 | 0.03 | 0.50 | 0.03 | 0.51 | 0.03 | 0.51 | 0.03 | 0.51 | 0.03 | 0.51 | 0.03 | 0.52 | 0.03 | 0.51 | 0.03 | 0.52 | 0.03 | 0.52 | 0.03 |
| SLFL | 0.55 | 0.03 | 0.54 | 0.03 | 0.55 | 0.03 | 0.54 | 0.03 | 0.55 | 0.03 | 0.55 | 0.03 | 0.55 | 0.03 | 0.54 | 0.03 | 0.55 | 0.03 | 0.55 | 0.03 |
