# Supplementary material for: Associations of White Matter Microstructure with Clinical and Demographic Characteristics in Heavy Drinkers
Source: PLoS One. 2015 Nov 3;10(11):e0142042. doi: 10.1371/journal.pone.0142042 (PMC4631485; doi:10.1371/journal.pone.0142042)
Supplement: S2 Table — (PDF) [file pone.0142042.s003.pdf]

S2 Table. Variance-Covariance Matrix for Confirmatory Factor Analysis of the White Matter Factor (WMF).

	Body of corpus callosum	Fornix	External capsule, right	External capsule, left	Cingulate gyrus, right	Cingulate gyrus, left	Superior longitudinal fasciculus, right	Superior longitudinal fasciculus, left
Body of corpus callosum	9.684	____	____	____	____	____	____	____
Fornix	10.207	47.541	____	____	____	____	____	____
External capsule, right	2.636	3.634	5.583	____	____	____	____	____
External capsule, left	2.446	2.175	4.734	6.562	____	____	____	____
Cingulate gyrus, right	4.535	7.315	4.064	3.822	12.379	____	____	____
Cingulate gyrus, left	5.197	7.816	4.158	3.941	9.960	12.784	____	____
Superior longitudinal fasciculus, right	3.362	4.953	3.693	2.980	5.488	5.523	8.081	____
Superior longitudinal fasciculus, left	3.452	3.861	3.642	3.256	5.307	5.490	6.329	7.467
